# Role of Natural and Synthetic Compounds in Modulating NRF2/KEAP1 Signaling Pathway in Prostate Cancer

**DOI:** 10.3390/cancers15113037

**Published:** 2023-06-02

**Authors:** Giovanni Tossetta, Sonia Fantone, Daniela Marzioni, Roberta Mazzucchelli

**Affiliations:** 1 Department of Experimental and Clinical Medicine, Università Politecnica delle Marche, 60126 Ancona, Italy; s.fantone@pm.univpm.it (S.F.); d.marzioni@univpm.it (D.M.); 2 Department of Biomedical Sciences and Public Health, Section of Pathological Anatomy, Università Politecnica delle Marche, 60126 Ancona, Italy; r.mazzucchelli@staff.univpm.it

**Keywords:** NRF2, chemotherapy, prostate cancer, signaling, compounds, KEAP1, antioxidants, natural, synthetic

## Abstract

**Simple Summary:**

Several studies showed that oxidative stress is involved in cancer occurrence, development, progression chemoresistance and radio resistance. The nuclear factor erythroid 2-related factor 2 (NRF2)/KEAP1 (Kelch-Like ECH-Associated Protein 1) pathway plays a key role in protecting cells against oxidative damage. Moreover, several studies demonstrated that both natural and synthetic compounds can modulate NRF2/KEAP1 signaling in normal and cancer cells. In this review, we discussed the current literature regarding the role of natural and synthetic compounds in regulating NRF2/KEAP1 signaling pathway in prostate cancer.

**Abstract:**

Prostate cancer is the second most common cancer in men worldwide. Prostate cancer can be treated by surgery or active surveillance when early diagnosed but, when diagnosed at an advanced or metastatic stage, radiation therapy or androgen-deprivation therapy is needed to reduce cancer progression. However, both of these therapies can cause prostate cancer resistance to treatment. Several studies demonstrated that oxidative stress is involved in cancer occurrence, development, progression and treatment resistance. The nuclear factor erythroid 2-related factor 2 (NRF2)/KEAP1 (Kelch-Like ECH-Associated Protein 1) pathway plays an important role in protecting cells against oxidative damage. Reactive oxygen species (ROS) levels and NRF2 activation can determine cell fate. In particular, toxic levels of ROS lead physiological cell death and cell tumor suppression, while lower ROS levels are associated with carcinogenesis and cancer progression. On the contrary, a high level of NRF2 promotes cell survival related to cancer progression activating an adaptive antioxidant response. In this review, we analyzed the current literature regarding the role of natural and synthetic compounds in modulating NRF2/KEAP1 signaling pathway in prostate cancer.

## 1. Introduction

Prostate cancer (PCa) is the second most common cancer in men [1,2]. Localized prostate cancer can be managed with active surveillance or prostatectomy if it is diagnosed early, whereas if it is diagnosed in advanced or metastatic stages, the current treatment is radiation therapy or androgen deprivation therapy (ADT) to reduce cancer progression [2,3,4].

Radiation induces DNA damage and vascular remodeling causing tumor hypoxia and leading to tumor cells apoptosis and necrosis [5]. ADT acts by reducing serum androgens and inhibiting androgen receptor, responsible for PCa development and growth. However, PCa resistance has been reported in both these therapies.

Resistance to radiation can be due to an increased level of DNA repair mechanisms and to an increase in the intracellular levels of reactive oxygen species (ROS). In fact, persistent oxidative stress can regulate various transcription factors/activators, such as nuclear factor (erythroid-derived 2)-related factor 2 (NRF2), that is recognized as a key feature for protecting cells from apoptosis and other mechanisms leading to the radioresistant phenotype [6,7].

Castration-resistant prostate cancer (CRPC) development is a consequence of long-term ADT [8,9]. CRPC is treated with second generation taxane such as cabazitaxel, but resistance to this chemotherapeutic can also occur [10,11]. CRPC onset may be due to androgen receptor gene mutations and amplification that causes a persistent androgen receptor signaling activation during ADT, enhancing tumor cells growth [12,13,14]. Moreover, the development of CRPC seems to be related to oxidative stress that is implicated in regulation of androgen receptor expression [15,16].

Oxidative stress is characterized by elevated intracellular levels of ROS, highly reactive molecules such as superoxide (O_2_^−^), hydroxyl radical (OH^−^) and hydrogen peroxide (H_2_O_2_) that can damage several cellular components including DNA, proteins and lipids [17,18]. NRF2 is a basic leucine zipper transcription factor that regulates the expression of several antioxidant enzymes by binding the antioxidant response elements (AREs) regions in the promoter of these genes [19,20]. Normally, NRF2 is bound to Kelch-Like ECH-Associated Protein 1 (KEAP1; its negative regulator) that targets NRF2 for proteasomal degradation. Under oxidant stimuli, ROS bind to cysteine residues of KEAP1 causing a conformational change that, inhibiting NRF2 ubiquitination and degradation, allows NRF2 nuclear translocation and its binding to the AREs of the antioxidant genes activating their expression [19,21].

NRF2/KEAP1 signaling pathway is involved in regulating oxidative stress conditions [22,23], which play a key role in inducing inflammation [17,24,25], endothelial disfunction [24,26,27] and cancer [17,21,28].

In fact, high ROS levels lead to cell death while low levels are associated to carcinogenesis and cancer progression. On the contrary, high levels of NRF2 can counteract ROS effects maintaining redox homeostasis and favoring cell survival [29]. Moreover, ROS levels are more frequently increased in cancer cells than in normal cells [30,31]. This can be due to the higher metabolism of cancer cells compared to normal cells [32].

Thus, the NRF2/KEAP1 pathway is an emerging chemo- and radio-therapeutic target in several cancer types including prostate cancer [7,19,21,33]. In fact, it has been reported NRF2 expression was significantly higher in chemo- and radio-resistant cancer tissues protecting cancer cells against the oxidative damage induced by chemotherapeutics and radiation therapy [19,33,34,35]. In addition, it has been reported that NRF2 can induce the expression of ATP-binding cassette (ABC) transporters that further protect cancer cells from chemotherapeutics pumping the drug out of the cells [34]. Moreover, NRF2 plays a key role in cancer stem cells (CSCs) survival and self-renewal, favoring their tumorigenicity and chemoresistance capacity [19].

For these reasons, natural and synthetic compounds able to decrease NRF2 expression in cancer cells can significantly improve the efficiency of the treatments ameliorating the outcome of the disease.

In this review, we analyzed the current literature regarding the role of natural and synthetic compounds on NRF2/KEAP1 pathway modulating ROS and/or NRF2 status in prostate cancer cells. In addition, we report new insight in the use of these compounds alone as chemo preventive agents or in combination with ADT or radiotherapy to evaluate a possible role of these compounds in improving the outcome of patients with prostate cancer.

## 2. NRF2 Modulation by Natural Compounds

Natural compounds are biological substances that can be found in plants (e.g., carotenoids, polyphenols, anthocyanins and flavonoids), bacteria, fungi and marine organisms [36,37]. These compounds are widely used as food supplements and showed important anti-inflammatory, antioxidant and anti-cancer effects [36,38,39,40,41]. Hormone and metabolic compounds can also interact with oxidative metabolism affecting tumoral cell growth [42,43,44].

### 2.1. Vegetable Substances

Ursolic acid (UA) is a triterpenoid phytochemical found in various fruits and vegetables [45] with anticancer, antioxidant and anti-inflammatory effects [46,47,48]. Interestingly, Li and colleagues showed that UA treatment attenuated the growth of xenografted human Vertebral-Cancer of the Prostate (VCaP) prostate cancer cells. Moreover, UA activated NRF2 pathway proving an important antioxidant capacity of this compound. However, the activation of NRF2 pathway by UA did not prevent cancer cell death [49]. This activation may be due to the alteration of methylation status of NRF2 gene promoter since the same effect was found in Transgenic Adenocarcinoma of the Mouse Prostate (TRAMP)-C1 cells treated with the UA analogue 2α-hydroxyursolic acid [28].

Phenethyl isothiocyanate is a natural compound present in cruciferous vegetables such as broccoli, brussels sprouts and cabbages with important antioxidant and anti-cancer effects [50,51,52]. Xu and colleagues found that phenethyl isothiocyanate treatment of PC3 cells significantly increased the phosphorylation of the mitogen-activated protein kinases (MAPK), extracellular signal-regulated kinases 1/2 (ERK1/2) and c-Jun NH2-terminal kinase 1/2 (JNK1/2), and induced the release of NRF2 from sequestration by KEAP1 favoring NRF2 translocation into the nucleus [53]. Importantly, phenethyl isothiocyanate-induced activation of ERK and JNK signaling significantly increased HO-1 expression. Interestingly, researchers also found that both ERK2 and JNK1 could directly phosphorylate NRF2 allowing its translocation into the nucleus activating HO-1 expression [53]. These results agree with a previous study reporting that MAPK can activate NRF2 signaling [54,55,56]. Moreover, both UA and phenethyl isothiocyanate affect the NRF2 signaling pathway inducing SET domain-containing lysine methyltransferase 7 (SETD7) expression and protecting DNA from oxidative damage [57]. In fact, NRF2 pathway in prostate cancer cells can be regulated by SETD7, a lysine methyltransferases that can add methyl groups to the lysine 4 on histone H3 (H3K4) favoring transcriptional activation [58,59,60]. In fact, knocking-down SETD7 in Lymph Node Carcinoma of the Prostate (LNCaP) and PC-3 cells led to a decreased expression of downstream NRF2 targets such as NAD(P)H: quinone oxidoreductase 1 (NQO1) and glutathione S-transferase theta 2 (GSTT2) increasing ROS levels. This effect was due to H3K4me1 enrichment reduction on the NRF2 and GSTT2 promoter regions causing NRF2 and GSTT2 decreased expression in SETD7 knockdown cells [57].

Sulforaphane is another natural compound belonging to the isothiocyanate group, widely present in the cruciferous vegetables, with known anticarcinogenic and anticancer activity [61]. It is known that sulforaphane increases NRF2 activity causing a decrease in full-length androgen receptor (AR-FL) expression in prostate cancer cell lines sensible to ADT [62]. In addition, sulforaphane inhibited the expression of both AR-FL and its splice variant AR-V7 in 22Rv1 cells [63].

Sulforaphane also showed interesting effects in radiotherapy increasing NRF2, HO-1, NQO1 and thioredoxin 6 (Trx6) expression, decreasing basal ROS levels and sensitizing 22RV1 cells to radiation [64].

Parthenolide is a sesquiterpene lactone present in the medical plant feverfew (*Tanacetum parthenium*) with important anti-inflammatory [65] and anti-cancer [66] effects. In addition, this natural compound seems to have a role in radiosensitivity. In fact, an interesting study showed that parthenolide can increase radiosensitivity of mouse xenograft tumors and protected normal prostate tissue against radiation-induced injury. Moreover, parthenolide increased ROS levels causing the oxidation of thioredoxin (TrX) in a LNCaP, PC3 and Duke University 145 (DU145) prostate cancer cells leading to a TrX-dependent increase on the reduction in KEAP1. In turn, KEAP1 induced mitochondrial phosphoglycerate mutase 5 (PGAM5) expression, a protein serine/threonine phosphatase that degrades Bcl-xL (BCL2L1) present in the mitochondrial membrane [67], inducing apoptosis of prostate cancer cells. In contrast, parthenolide treatment significantly increased oxidation of KEAP1 in normal prostate epithelial cells (prostate epithelial viral transformed PZ-HPV-7 (PZ) and RWPE-1 cell lines) increasing NRF2 levels with a consequent increase in the expression of NRF2-dependent antioxidant enzymes such as Manganese Superoxide Dismutase (MnSOD) and Copper-zinc-superoxide dismutase (CuZnSOD) [68]. This study showed that parthenolide can differently modify the redox state of KEAP1 in tumor and normal prostate cells suggesting a possible use of this compound as tumor-specific radiosensitizing agent with radioprotective properties in normal cells.

Polyphenol-rich fraction of *Bergenia ligulata* (PFBL) is a natural compound widely used in Indian traditional and folk medicine due to its anti-inflammatory and antineoplastic properties [69]. In in vitro study, PFBL treatments induced cell apoptosis in androgen-dependent LNCaP and androgen-refractory PC3, DU145 and TRUMP-C1 cells. Researchers showed that PFBL acted enhancing catalytic activity of monoamine oxidase A (MAO-A) with consequently upregulation of ROS production. Moreover, PFBL significantly inhibited NRF2, Glutathione peroxidase (GPx), SOD1 and catalase expression promoting PC3 cell death. Thus, PFBL exerts its anticancer activity inhibiting cell antioxidant capacity [70].

Puerarin is an isoflavone-C-glycoside widely used in traditional Chinese medicine due to its anticancer, anti-inflammatory and antioxidants activities [71,72,73]. It has been reported that treatment with puerarin of androgen-independent (DU145 and PC-3) and androgen-dependent LNCaP prostate cancer cells significantly decreased cell viability. Moreover, DU145 and PC-3 exposure to puerarin increased intracellular ROS and Lactate dehydrogenase (LDH) production leading to apoptosis. Furthermore, puerarin increased KEAP1 protein expression and decreased NRF2, HO-1 and NQO1 protein expression in DU145 and PC3 cells [74]. Thus, puerarin exerts its anticancer effect in prostate cancer cells by inhibiting cells antioxidant capacity.

Boric acid is abundant in vegetables, nuts, legumes and fruit, and its intake is associated with reduced risk of prostate cancer [75,76,77]. It has been reported that the translocation of NRF2 from the cytoplasm to the nucleus requires NRF2 phosphorylation, which is a protein kinase R-like endoplasmic reticulum kinase (PERK)-dependent mechanism [78]. DU-145, Mouse Embryonic Fibroblasts (MEFs) wild type and PERK-deficient MEF (MEF PERK^−/−^) cell lines treated with boric acid showed that NRF2 was translocated into the nucleus in DU-145 and wild type MEFs but not in the MEFs PERK^−/−^ cells. Moreover, boric acid treatment increased HO-1, NQO1, and Glutamate-Cysteine Ligase Catalytic Subunit (GCLC) expression in DU-145 cells while increased HO-1 and GCLC in MEF WT cells [79]. This study showed a clear role of boric acid as an important antioxidant agent and demonstrated a key role of PERK in NRF2 translocation into the nucleus.

Camptothecin is a natural cytotoxic alkaloid originally isolated from *Camptotheca acuminate* which possesses potent anti-inflammatory, anti-cancerous, and anti-proliferative effects [80,81]. It has been reported that camptothecin treatment significantly inhibited proliferation and invasion of DU145 cells. Moreover, the authors found that camptothecin inhibited phorbol-12-myristate-13-acetate (PMA)-induced matrix metalloproteinase-9 (MMP-9) and vascular endothelial growth factor (VEGF) expression by NRF2 activation (nuclear translocation) increasing HO-1 expression. In fact, it has been reported that HO-1 can directly attenuate MMP-9 and VEGF production [82]. Thus, camptothecin may be a good candidate to inhibit prostate cancer progression [83].

The green algae of the genus *Ulva* (known as sea lettuce) are commonly consumed seaweeds with reported anti-inflammatory and antitumoral properties [84,85,86,87]. Interestingly, *Ulva* sp. extract treatment activated NRF2 pathway in LNCaP prostate cancer cells increasing NQO1 mRNA expression suggesting an important role of this seaweed in modulating NRF2 signaling [88].

Several studies reported the healthful effects of soy products (e.g., soybeans, soymilk products and soy flour) stating a lower risk of cancer in subjects consuming these products [89,90,91,92,93]. These protective effects may be associated to the highest levels of isoflavones that can be found in these products. In fact, isoflavones have important anti-oxidant and anti-inflammatory effects [90,94,95]. An interesting study by Barve and colleagues evaluated the effect of soy isoflavones in the prostates of NRF2 knockout and wildtype mice. These authors found that soy isoflavones activated NRF2-dependent genes. Moreover, they showed that genes modulated by soy isoflavone and regulated by NRF2 belonged to the categories of molecules concerning electron transport, phase II metabolizing enzymes, cell growth and differentiation, apoptosis, cell cycle, transcription factors, transport, mRNA processing and carbohydrate homeostasis. Thus, soy isoflavones may play an important role in prostate cancer chemoprevention [96].

Looking at the studies discussed in this section, we can conclude that many of the vegetable substances analyzed activate NRF2 increasing the expression of antioxidant enzymes such as SOD, NQO1 and HO-1. Moreover, these compounds can reduce AR, MMP-9 and VEGF expression favoring cancer cells sensitivity to chemo- and radiotherapy.

It is interesting to note that PFBL and puerarin decrease NRF2 expression directly inducing cell death.

### 2.2. Vitamins

Eight natural Vitamin E analogues have been found: α-, β- and γ-δ-tocopherol, and α-, β- and γ-δ-tocotrienol [28]. γ-tocopherol showed anti-hypertensive, antioxidant, anti-inflammatory and anticancer effects [28,97]. α-Tocopheryl succinate is one of the most effective analogues of vitamin E and it can significantly inhibit cell proliferation and induce cell death in several types of cancer cell lines [28,98]. The anti-cancer activity of α-Tocopheryl succinate may be due to its ability in inhibiting oxidative phosphorylation at the level of mitochondrial complexes I and II, enhancing ROS generation which, in turn, activate the expression of NRF2-dependent antioxidant genes [99,100]. However, this cytoprotective effect can increase resistance of PC3 prostate cancer cells to the oxidant damage induced by chemotherapeutics. Bellezza and colleagues found that a short-term (4 h) α-Tocopheryl succinate treatment of PC3 prostate cancer cell line significantly reduced cell viability increasing ROS production, but also intracellular glutathione (GSH) content [101]. Moreover, α-Tocopheryl succinate increased NRF2 nuclear translocation increasing the expression of HO-1 and decreasing NF-κB nuclear translocation. Interestingly, this effect was suppressed by the pharmacological inhibition of HO-1. Thus, α-Tocopheryl succinate can inhibit NF-κB activation upregulating HO-1 expression [101].

The same research group demonstrated that α-tocopheryl succinate pre-treatment of PC3 prostate cancer cell line significantly increased glutathione intracellular content when exposed to paraquat (1,1′-dimethyl-4,4′-bipyridinium), a widely used non-selective quinone herbicide that induces oxidative stress. Since quinones are detoxified by NQO1, whose expression is mainly regulated by NRF2/KEAP1 signaling [19,28,33], the authors evaluated whether this enzyme was modulated by α-tocopheryl succinate. They found that NQO1 was responsible for α-tocopheryl succinate-induced adaptive response, although NQO1 expression was not altered by α-tocopheryl succinate treatment. In fact, the authors found that NQO1 silencing or its activity inhibition by dicumarol counteracted the α-tocopheryl succinate-induced adaptive response. Thus, α-tocopheryl succinate can exert cytotoxic effects while inducing an adaptive response to pro-oxidant stimuli [102].

Vitamin C (also known as L-ascorbic acid or ascorbate) and quercetin (a plant flavonoid) are two natural compounds with anticancer activity that can be found in several fruits and vegetables [33,103,104,105,106]. Interestingly, Abbasi and colleagues showed that treatment with Vitamin C and Quercetin of PC3 and DU145 cells led to a significant reduction in NRF2 mRNA expression and protein levels accompanied by a significant decrease in GPx and NQO1 enzymatic activity [107].

Thus, while α-Tocopheryl increases NRF2 and HO-1 expression, vitamin C decreases NRF2, GPx, GR and NQO1 expression.

### 2.3. Trace Elements

Selenium is a trace element essential for reduction in oxidative stress and DNA repair, and it is a component of the active glutathione peroxidase (GPx) domain [108]. Conflicting evidence is present on the role of selenium as chemopreventive agent. In fact, several studies claim that selenium may reduce the incidence of cancer [109,110,111], while others, such as the Selenium and Vitamin E Chemoprevention Trial (SELECT), did not show any beneficial effect of selenium in preventing prostate cancer onset [112,113].

An interesting study evaluated the possible chemopreventive properties of two organoselenium compounds (3-selena-1-dethiacephem and 3-selena-1-dethiacephem) in human prostate cancer LNCaP cells. The authors found that these compounds strongly activated the NRF2 signaling (increased expression in the nuclei) increasing HO-1 expression and significantly reducing ROS levels, proving that these compounds have a strong antioxidant effect. It is important to note that both these compounds significantly inhibited cell growth [114]. Thus, it may be suggested that they could be used as pharmacological agents for chemoprevention of prostate cancer.

Methaneseleninic acid, a precursor of methylselenol [115], an important metabolite of selenium [116,117,118] showed similar effect to organoselenium compounds. An interesting study by Singh and colleagues found that methaneseleninic acid and γ-tocopherol treatment of nude Nu/J mice implanted with 22Rν1 cells significantly decreased tumor volume/weight and serum PSA level. Furthermore, the authors found that γ-tocopherol alone and in combination with methaneseleninic acid increased apoptosis and decreased NRF2 expression [119]. Thus, a combination of methaneseleninic acid and γ-tocopherol intake could have a beneficial effect in prostate cancer management.

Summarizing, organoselenium compounds activate NRF2 increasing HO-1 expression, reducing ROS levels and cell growth while methaneseleninic acid decreases NRF2 expression.

### 2.4. Microbial Compounds

Salinomycin is a polyether antibiotic isolated from Streptomyces albus strain (Strain No. 80614) with antiviral, anti-inflammatory and anti-cancer effects [120,121]. Yu and colleagues found that salinomycin inhibited the viability by inducing apoptosis in a dose-dependent manner increasing ROS production and lipid peroxidation in both DU145 and PC-3 prostate cancer cell lines. Moreover, salinomycin significantly downregulated NRF2, heme oxygenase-1 (HO-1), NQO1 and glutamate-cysteine ligase (GCL) expression. Furthermore, salinomycin treatment decreased the activity of the antioxidant enzymes such as superoxide dismutase (SOD), catalase (CAT) and glutathione peroxidase (GPx) in both PC-3 and DU145 cell lines proving that this compound may trigger apoptosis by inducing oxidative stress suppressing NRF2 signaling [122].

### 2.5. Hormones

Dihydrotestostearone (DHT) is a potent androgen derived from conversion of testosterone by 5-alpha reductase. DHT can bind to the androgen receptor (AR) playing a crucial role in growth and maintenance of prostate epithelial cells. DHT stimulates the androgen receptor-mediated transactivation of several genes, involved in prostate cancer cell growth, carrying an androgen response element (ARE) in their promoters [123]. DHT treatment of androgen-dependent (LNCaP) and castration-resistant LNCaP C4-2B prostate cancer cells induced the transactivation of the androgen response element (ARE) in both cell lines, but it was significantly greater in LNCaP C4-2B cells than in LNCaP cells [124]. DHT-induced androgen receptor transactivation was associated with higher nuclear translocation of the NRF1 isoform p65-NRF1 in LNCaP C4-2B cells, compared to LNCaP cells. p65-NRF1 silencing attenuated androgen receptor transactivation while p65-NRF1 overexpression enhanced androgen receptor transactivation. In addition, DHT treatment suppressed NRF2 expression in LNCaP C4-2B cells, while NRF2 was significantly increased in LNCaP cells. These authors also found that both p65-NRF1 and p120-NRF1 isoforms, but not NRF2, physically interacts with androgen receptor enhancing its DNA-binding activity. However, while p65-NRF1 has an activator function on androgen receptor, the isoform p120-NRF1 had an inhibitory effect on androgen receptor transactivation. Importantly, they found that NRF2 exerted a suppressive effect on androgen receptor transactivation by increasing nuclear p120-NRF1 levels [124]. Thus, CRPC cells may bypass the suppressive effects of NRF2 on androgen receptor transactivation during ADT selecting tumoral clones considering the decrease in NRF2 expression. NRF2 expression decreases in adults with age [125] and prostate cancer incidence is higher in aging men [126]; therefore, this study suggests an association between decreased NRF2 expression and more aggressive prostate cancer occurrence in the elderly.

Melatonin is a hormone produced by the pineal gland that regulates circadian rhythm, mood and sleep, but it also showed important oncostatic activity in several types of human cancer including prostate cancer [127,128,129,130]. Interestingly, it has been found that melatonin treatment of nude mice xenografted with LNCaP human prostate cancer cells significantly increased NRF2 expression in xenograft indicating a direct antiradical scavenging effect of melatonin. However, this antioxidant effect was not sufficient to increase xenograft growth rate, which was significantly lower in mice treated with melatonin due to an inhibition of angiogenesis (by melatonin) [131,132].

Thus, DHT induces NRF2 activation reducing AR transactivation by increasing nuclear p120-NRF1 levels while melatonin increased NRF2 expression indicating a direct antiradical scavenging effect.

### 2.6. Metabolic Compounds

It is known that high glucose concentrations induce the binding of glucose to proteins forming glycosylation end products and inducing oxidative stress [133,134]. It has been reported that treatment of LNCaP cells with high glucose concentration significantly reduced cell proliferation increasing apoptosis. Moreover, high glucose concentrations significantly increased the content of ROS, LDH and interleukin-6 (IL-6), but decreased the content of IL-10. Interestingly, high glucose concentrations treatment lowered the protein expression levels of NRF2, HO-1, and γ-glutamyl cysteine synthetase (γ-GCS) demonstrating that high glucose levels inhibit NRF2/KEAP1 signaling pathway in prostate cancer cells, increasing the content of ROS promoting the apoptosis of prostate cancer cells [135].

Radiotherapy is another common anticancer therapy for many types of cancer, including prostate cancer, especially in the case of metastasis occurrence [136,137]. It has been reported that patients with diabetes suffer from increased lymph node metastasis, tumor recurrence and decreased survival compared to non-diabetic prostate cancer patients [138]. This may be due to the oxidative stress in diabetes that causes a chronic low-grade inflammation [139,140,141], leading to an additional oxidative damage in patients under radiotherapy.

4-Hydroxynonenal is an end product of lipoperoxidation process showing antiproliferative and proapoptotic properties in several types of tumors [142,143,144]. An interesting study reported an increased sensitivity to 4-Hydroxynonenal in PC3 and LNCaP cells compared to DU145 cells. Moreover, 4-Hydroxynonenal treatment of DU145 cells did not induce ROS production, DNA damage and apoptosis, but generated a lower amount of 4-Hydroxynonenal-protein adducts, unlike PC3 and LNCaP cells. These effects were explained by demonstrating that DU145 cells had lower KEAP1 expression and an increased NRF2 expression, and a greater GSH and glutathione S-transferase alpha 4 (GSTA4) content compared to PC3 and LNCaP cells. NRF2 silencing resulted in a reduction in GST A4 expression and GS-HNE formation demonstrating that NRF2 regulates 4-Hydroxynonenal metabolism. In addition, NRF2 silencing increased the antiproliferative and proapoptotic activity of 4-Hydroxynonenal in DU145 cells [145]. Thus, an increased NRF2 expression/activity can represent a potential mechanism of resistance to pro-oxidant therapy since NRF2 can reduce 4-Hydroxynonenal sensitivity in prostate cancer cells.

We can conclude that high glucose levels decease NRF2, HO-1 and γ-GCS expression while high NRF2 expression in cancer cells make them resistant to 4-Hydroxynonenal treatment.

The studies discussed in this paragraph, and summarized in Table 1, clearly show that natural compounds can efficiently modulate NRF2/KEAP1 signaling in normal and prostate cancer cells.

## 3. NRF2 Modulation by Synthetic Compounds

Synthetic compounds are pharmacologically active molecules man-made by synthesis and formed by any chemical reaction, either by chemical synthesis (chemosyntesis) or by biosynthesis. In addition, they derived from original compounds by synthetic modification to get or to improve a specific activity. Both synthetic drugs and environmental toxicants interfere in the modulation of NRF2.

Halofuginone is a synthetic inhibitor of NRF2 signaling pathway [136,137]. Interestingly, Satoshi Endo and colleagues reported a higher NRF2 nuclear expression in cabazitaxel-resistant prostate cancer 22Rv1/Cab-R cells compared to the cabazitaxel sensitive 22Rv1 cells [146]. In addition, it has been reported an increased expression of NRF2/KEAP1 pathway and its downstream enzymes such as γ-glutamylcysteine synthetase (γ-GCS), Carbonyl reductase 1 (CBR1), NQO1 and HO-1 in 22Rv1/Cab-R cells. NRF2 silencing significantly increased cell cabazitaxel sensitivity in 22Rv1/Cab-R cells. Consequently, halofuginone treatment led to a decrease in the NRF2 downstream enzymes γ-GCS, CBR1, NQO1 and HO-1. Thus, NRF2 signaling plays a key role in carbaxitel resistance in prostate cancer [146].

Bardoxolone-methyl, also known as CDDO-Me or RTA 402, is a synthetic triterpenoid antioxidant drug with anti-inflammatory and anticancer activity [147,148,149]. It has been reported that Bardoxolone-methyl can activate NRF2 pathway by binding KEAP1 then impeding NRF2 ubiquitination and degradation [150]. An interesting study found that Bardoxolone-methyl rapidly downregulated the full-length androgen receptor (AR-FL) in LNCaP and C4-2B cells, and both AR-FL and its splice variant AR-V7 in 22Rv1 cells. In addition, Bardoxolone-methyl significantly increased NRF2 decreasing ROS production in 22Rv1 cells, demonstrating the important antioxidant capacity of this compound [151].

Synthetic 5α-reductase inhibitors such as finasteride and durasteride gained significant interest as possible prostate chemopreventive agents. In fact, two clinical trials showed that finasteride and durasteride significantly reduced the incidence of prostate cancer formation in men [152,153]. However, these studies showed that the occurrence of aggressive prostate tumors (Gleason scores 7–10) was increased in the patients given finasteride or durasteride [152,153]. An interesting study by Yun and colleagues found that finasteride treatment of androgen-refractory PC3 cells did not alter cell growth or apoptosis. However, finasteride induced the expression of NRF2 and HO-1. Notably, the basal expression level of NRF2 protein was higher in androgen-refractory prostate cancer cells (DU-145 and PC-3 cells) when compared with androgen-responsive prostate cancer cells (LNCaP cells). Furthermore, finasteride selectively induced NRF2 expression in DU-145 and PC-3 cells, but not in LNCaP cells. Thus, finasteride-mediated induction of NRF2 might contribute to the development of high-grade prostate tumor formation in men since NRF2 can favor cancer cells survive to chemotherapeutics [154].

The androgen receptor plays a key role in prostate cancer development and progression; therefore, the inhibition of its expression/function could significantly improve the therapeutic approach against prostate cancer [155,156,157].

Curcumin (1,7-bis(4-hydroxy-3-methoxyphenyl)-1,6-heptadiene-3,5-dione) is a polyphenolic compound present in the rhizomes of *Curcuma longa* Linn. with proved anti-inflammatory, anti-oxidant and anti-cancer properties [36,158,159]. It is interesting to note that synthetic analogues of curcumin showed a stronger anti-cancer activity than natural curcumin [160,161]. Thus, researchers aimed to synthetize these compounds in order to evaluate their potential role in treatment of several pathologies including prostate cancer [28,160,161,162,163].

The curcumin analogue 27 (ca27) is a symmetrical diphenolic analogue of curcumin which, in contrast to curcumin, has a shorter 5-carbon unsaturated linker with a single carbonyl group [164]. An interesting study found that ca27 treatment significantly decreased AR protein expression in LNCaP, C4-2, and LAPC-4 cells. Moreover, ca27 induced ROS formation, but also NRF2 activation and NQO1 and aldoketoreductase 1C1 (AKR1C1) expression. Interestingly, AR protein loss was preceded by ROS production and ca27-mediated down-regulation of the AR was decreased when cells were treated with N-acetyl cysteine, an antioxidant compound [165]. Thus, ca27 induces ROS production and downregulates AR by an oxidative stress mechanism, suggesting that ca27 could be a good anti-androgenic compound to treat patients with advanced prostate cancer.

Inorganic arsenic is a ubiquitously distributed environmental and industrial toxicant with potential carcinogenic capacity. Thus, people chronically exposed to this environmental toxicant have an increased risk of prostate cancer [166,167,168]. In fact, it has been reported that inorganic arsenic may induce prostate carcinogenesis transforming normal human prostate stem-progenitor cells (PrSPCs).

PrSPCs treated with low-dose inorganic arsenic showed an increased self-renewal and suppressed differentiation. Moreover, transcriptomic analysis showed an activation of oncogenic pathways as the NRF2/KEAP1 pathway in PrSPCs when these were exposed to inorganic arsenic. NRF2 activation led to an increased expression of its downstream enzymes such as NQO1, HO-1, Thioredoxin Reductase 1 (TXNRD1), Glutathione Peroxidase 2 (GPX2), Aldo-keto reductase family 1 member C3 (AKR1C3) and Ubiquitin C-Terminal Hydrolase L1 (UCHL1) [169]. Interestingly, the authors found that NRF2 knockdown suppressed PrSPC differentiation revealed by spheroid growth inhibition, whereas its activation enhanced spheroid growth. Autophagy is a conserved cellular recycling process by which cytoplasmic organelles, proteins or others cytosolic components are sequestered in a double-membrane-bound autophagosome and targeted to lysosomal degradation [169]. It has been shown that autophagy plays a pivotal role in tumor progression and drug resistance by helping tumor cells to manage intracellular and environmental stress surviving to many detrimental oxidative processes then leading to therapy resistance [170]. Inorganic arsenic also inhibited the autophagic protein degradation of p62, an activator of NRF2 [171,172]. Thus, inorganic arsenic activates NRF2 through autophagic flux blockade in prostate progenitor cells [173].

MnTE-2-PyP (Manganese (III) Meso-Tetrakis-(N-Methylpyridinium-2-yl) porphyrin) is a manganese porphyrin with antioxidant effects [174]. It has been reported that MnTE-2-PyP can decrease tumor volume and increase survival in a prostate cancer mouse model [175].

Interestingly, MnTE-2-PyP treatment of human prostate fibroblast cells in an irradiated hyperglycemic environment protected against hyperglycemia-induced cell death after radiation. This cytoprotective effect was due to the increased expression of NRF2 and NQO1 in these cells. Thus, MnTE-2-PyP can protect fibroblasts from irradiation and hyperglycemia damage activating NRF2 pathway in diabetic prostate cancer patients undergoing radiotherapy [176]. Another study demonstrated that MnTE-2-PyP treatment of mouse primary prostate fibroblast cells increased NRF2, SOD2 and NQO1 expression downregulating KEAP1 expression. Interestingly, activation of the NRF2 pathway was essential to prevent myofibroblast formation, a key process responsible for the fibrotic phenotype in prostate tissue during radiation exposure. Thus, MnTE-2-PyP-mediated activation of NRF2 may play a key role in preventing fibrosis in prostate tissues during radiotherapy [177].

Clofibrate is an amphipathic carboxylic acid normally used for managing the high cholesterol and triacylglyceride levels in blood, but it can also activate the nuclear receptor peroxisome proliferator activatingreceptor alpha (PPARα) which can induce apoptosis in cancer cells [19]. It has been demonstrated that clofibrate can induce HO-1 expression in a concentration- and time-dependent manner. Interestingly, research found that the increased HO-1 expression was due to NRF2 signaling pathway activation but not to PPARα pathway activation. In fact, NRF2 silencing significantly attenuated clofibrate-induced HO-1 gene transcription while silencing of PPARα had no effect on clofibrate-induced HO-1 promoter activity in DU145 cells. Thus, clofibrate can induce HO-1 gene expression through a PPARα-independent mechanism while NRF2 signaling pathway is essential for HO-1 induction [178].

Pterocarpanquinone is a synthetic compound with anti-inflammatory and anti-cancer effects [179,180,181,182]. Treatment of PC3, LNCaP and Los Angeles Prostate Cancer-4 (LAPC4) prostate cancer cell lines with pterocarpanquinone significantly increased ROS production and lipid peroxidation inducing apoptosis. However, pterocarpanquinone treatment also increased NRF2, NQO1 and SOD expression [183]. Thus, the increased ROS levels induced by pterocarpanquinone tend to be counteracted by activating NRF2 signaling, but this antioxidant response was not sufficient to avoid cell death.

Adenosine is a nucleoside that can bind specific receptors [184,185] present in several tissues, modulating important functions such as neurotransmitter release, vasoconstriction or vasodilation of veins and arteries, T cell proliferation, cytokines production and bronchoconstriction [184]. In addition, it has been demonstrated that adenosine and its analogues can trigger apoptosis of various cell types inhibiting cell growth [184,186]. Minelli and colleagues reported that 2-Chloroadenosine (an adenosine analogue) significantly reduced viability and increased apoptosis in both androgen-independent and -sensitive (PC3 and LNCaP) prostate cancer cell lines. Moreover, 2-Chloroadenosine significantly reduced GSH content and increased ROS levels in PC3 whereas only ROS production was increased in LNCaP cells. It is important to note that 2-Chloroadenosine treatment increased NRF2 nuclear translocation in PC3 cells confirming that these cells were oxidatively stressed by 2-Chloroadenosine treatment. In addition, this antioxidant defense mechanism was not sufficient to counteract oxidative stress induced by 2-Chloroadenosine treatment [187].

Looking at the studies discussed in this section, we can conclude that many of the synthetic compounds analyzed activate NRF2 increasing the expression of antioxidant enzymes such as NQO1, SOD2, GPX2 and HO-1. However, halofuginone decreased NRF2, NQo1, HO-1 and γ-GCS expression in cabazitaxel-resistant 22Rv1/Cab-R cells.

It is interesting to note that ca27 induced NRF2 activation decreasing AR expression. Thus, this compound is also involved in NRF2/AR axis.

The studies discussed in this paragraph, and summarized in Table 2, show that synthetic compounds can also modulate NRF2/KEAP1 signaling in normal and prostate cancer cells.

## 4. Conclusions

**Figure 1:**
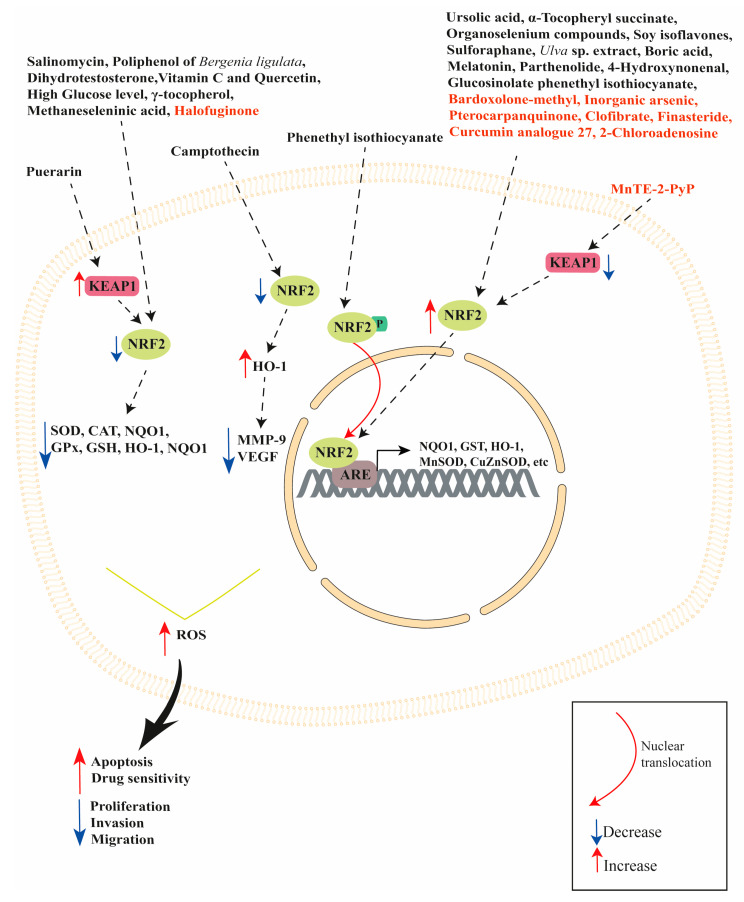
Schematic representation of NRF2/KEAP1 signaling pathway modulation by natural (in black) and synthetic (in red) compounds.

The NRF2/KEAP1 signaling pathway is a promising target in the treatment of prostate cancer since it plays an important role in prostate cancer onset, progression and treatment resistance. In this review, we discussed several studies focused on the multifaceted role of NRF2/KEAP1 signaling pathway in regulating several cell processes, including antioxidant response, cell proliferation, apoptosis and autophagy. Analyzing the current literature, it was revealed that NRF2/KEAP1 signaling pathway can be modulated by natural and synthetic compounds (see Table 1 and Table 2) in both in vivo and in vitro models of prostate cancer. A schematic representation of the NRF2/KEAP1 signaling pathway modulation by natural and synthetic compounds is shown in Figure 1.

New insights in the use of these compounds alone as chemopreventive agents or in combination with androgen deprivation therapy or radiotherapy were reported in order to clarify if they could have a clinical impact in prostate cancer care. We reported that these compounds can act directly, changing NFR2 status, or indirectly, inducing ROS increase.

Thus, both natural and synthetic compounds could be used in clinical practice to improve the outcome of patients affected by prostate cancer.

## Figures and Tables

**Table 1:** NRF2/KEAP1 signaling modulation by natural compounds.

Modulator	Model Studied	Effect	Reference
Ursolic acid (UA)	VCaP cells	UA activated NRF2 pathway	[49]
Phenethyl isothiocyanate (PEITC)	PC3 cells	PEITC increased ERK1/2 and JNK1/2 phosphorylation. Phenethyl isothiocyanate induced NRF2 phosphorylation favoring its translocation into the nucleus, which increased HO-1 expression. This effect was attenuated when ERK and JNK signaling was inhibited. Both ERK2 and JNK1 can directly phosphorylate NRF2.	[53]
PEITC and UA	LNCaP and PC3 cells	PEITC and UA treatment induced SETD7 expression activating NRF2 signaling pathway leading to increased NQO1 and GSTT2 mRNA expression, protecting DNA from oxidative damage.	[57]
Sulforaphane	22RV1 cells	Sulforaphane inhibited both AR-FL and AR-V7 expression. NRF2 induction (through the NRF2 activator bardoxolone methyl) significantly decreased both AR-FL and AR-V7 levels.	[63]
Sulforaphane	22RV1 cells	Sulforaphane increased NRF2, HO-1, NQO1 and Trx6 expression, decreasing basal ROS levels and sensitizing cells to radiation.	[64]
Parthenolide	LNCaP, PC3, DU145, PZ and RWPE-1 cell lines	Parthenolide increased ROS levels in LNCaP, PC3 and DU145 prostate cancer cells, leading to apoptosis. Parthenolide increased oxidation of KEAP1 in normal prostate PZ and RWPE-1 cell lines, increasing NRF2, MnSOD and CuZnSOD expression.	[68]
Polyphenol-rich fraction of *Bergenia ligulata* (PFBL)	LNCaP, PC3, DU145 and TRUMP-C1 cells	PFBL treatments induces cell apoptosis enhancing catalytic activity of MAO-A, consequently upregulating ROS production. PFBL inhibited NRF2, GPx, SOD1 and catalase expression, promoting PC3 cell death.	[70]
Puerarin	androgen-independent (DU145 and PC-3) and androgen-dependent LNCaP cells	Puerarin decreased cell viability in DU145, PC-3 and LNCaP cells. DU145 and PC-3 exposure to puerarin increased apoptosis, intracellular ROS and LDH production. Puerarin increased KEAP1 protein expression and decreased NRF2, HO-1 and NQO1 protein expression in DU145 and PC3 cells.	[74]
Boric Acid	DU145, MEFs wild type and MEFs PERK^−/−^	Boric acid induced NRF2 translocation into the nucleus in DU-145 and wild type MEFs, but not in the MEFs PERK-/- cells. Boric acid increased the expression of HO-1, NQO1 and GCLC in DU-145 cells, and HO-1 and GCLC in MEF WT cells.	[79]
Camptothecin	DU145 cells	Camptothecin inhibited cell proliferation and invasion. Camptothecin inhibited PMA-induced MMP-9 and VEGF expression by NRF2 activation and HO-1 expression, which directly attenuates MMP-9 and VEGF production.	[83]
*Ulva* sp. extract	LNCaP	*Ulva* sp. extract treatment activated NRF2 pathway increasing NQO1 mRNA expression.	[88]
Soy isoflavones	NRF2 knockout and wildtype mice.	Soy isoflavones induced NRF2 expression while NRF2 knockout altered the expression of genes involved in electron transport, phase II metabolizing enzymes, cell growth and differentiation, apoptosis, cell cycle, transcription factors, transport, mRNA processing and carbohydrate homeostasis.	[96]
α-Tocopheryl succinate	PC3 cells	α-Tocopheryl succinate reduced cell viability increasing ROS production and intracellular GSH content. α-Tocopheryl succinate increased NRF2 nuclear translocation and HO-1 expression, while decreasing NF-κB nuclear translocation. α-Tocopheryl succinate inhibited NF-κB activation upregulating HO-1 expression. α-Tocopheryl increased glutathione intracellular content when exposed to the oxidant paraquat. α-tocopheryl succinate did not alter NQO1 expression, but NQO1 silencing or its activity inhibition by dicumarol counteracted the α-tocopheryl succinate-induced adaptive response.	[101,102]
Vitamin C Quercetin	PC3 and DU145 cells	Vitamin C and Quercetin cotreatment reduced NRF2 mRNA and protein expression accompanied by a decrease in GPx, GR and NQO1 enzymatic activity.	[107]
Organoselenium compounds	LNCaP cells	Organoselenium compounds activated NRF2 (increased expression in the nuclei) increasing HO-1 expression, reducing ROS levels and cell growth.	[114]
Methaneseleninic acid and γ-tocopherol	22Rν1 cells implanted in nude Nu/J mice	Methaneseleninic acid and γ-tocopherol decreased tumor volume/weight and serum PSA. γ-tocopherol alone and in combination with methaneseleninic acid increased apoptosis and decreased NRF2 expression.	[119]
Salinomycin	DU145 and PC-3 cells	Salinomycin inhibited cell viability and induced apoptosis in both cell lines increasing ROS and decreasing NRF2, HO-1, NQO1 and GCL expression. Salinomycin decreased the activity of SOD, CAT, and GSH-Px enzymes.	[122]
Dihydrotestosterone (DHT)	LNCaP and LNCaP C4-2B cells	DHT treatment induced ARE transactivation in both cell lines, but it was greater in LNCaP C4-2B than in LNCaP cells. DHT-induced androgen receptor transactivation was associated with higher nuclear translocation of p65-NRF1 in LNCaP C4-2B cells compared to LNCaP cells. p65-NRF1 silencing attenuated androgen receptor transactivation, while p65-NRF1 overexpression enhanced androgen receptor transactivation. Conversely, DHT treatment completely suppressed NRF2 expression in LNCaP C4-2B cells, while NRF2 was significantly increased in LNCaP cells. p65-NRF1 and p120-NRF1 isoforms, but not NRF2, physically interacts with androgen receptor enhancing its DNA-binding activity. p65-NRF1 has an activator function on androgen receptor, while p120-NRF1 had an inhibitory effect on androgen receptor transactivation. NRF2 exerted a suppressive effect on androgen receptor transactivation by increasing nuclear p120-NRF1 levels.	[124]
Melatonin	LNCaP xenografted in nude mice	Increased NRF2 expression in xenograft.	[131,132]
High glucose concentrations	LNCaP	High glucose levels reduced cell proliferation increasing apoptosis, ROS, LDH and interleukin-6 (IL-6), but decreased the content of IL-10. High glucose levels lowered NRF2, HO-1 and γ-GCS expression.	[135]
4-Hydroxynonenal	PC3, LNCaP and DU145 cells.	PC3 and LNCaP cells are more sensitive to 4-Hydroxynonenal compared to DU145 cells. Different from PC3 and LNCaP cells, 4-Hydroxynonenal did not induce ROS production, cause DNA damage or generate a lower amount of 4-Hydroxynonenal-protein adducts and did not induce apoptosis in DU145 cells. DU145 cells had lower KEAP1 expression and an increased expression of NRF2 with greater GSH and GST A4 content compared to PC3 and LNCaP cells. NRF2 silencing reduced GST A4 expression and GS-HNE formation, increasing the antiproliferative and proapoptotic activity of 4-Hydroxynonenal in DU145 cells.	[145]

**Table 2:** NRF2/KEAP1 signaling modulation by synthetic compounds.

Modulator	Model Studied	Effect	Reference
Halofuginone	cabazitaxel-resistant 22Rv1/Cab-R and cabazitaxel-sensitive 22Rv1 cells	Halofuginone increased cabazitaxel sensitivity, suppressing NRF2 and its downstream enzymes γ-GCS, CBR1, NQO1 and HO-1 expression in 22Rv1/Cab-R cells.	[146]
Bardoxolone-methyl	22RV1 cells	Bardoxolone-methyl increased NRF2 expression, decreasing ROS production.	[151]
Finasteride	DU-145, PC-3 and LNCaP cells	Finasteride increased NRF2 and HO-1 expression. Basal expression level of NRF2 protein was higher in androgen-refractory prostate cancer cells (DU-145 and PC-3 cells) compared to androgen-responsive prostate cancer cells (LNCaP cells). Finasteride selectively induced the expression of NRF2 in DU-145 and PC-3 cells, but not in LNCaP cells.	[154]
Curcumin analogue 27 (ca27)	LNCaP, LNCaP C4-2, and LAPC-4 cells.	ca27 decreased androgen receptor expression and induced ROS formation, but also induced NRF2 activation, NQO1 and AKR1C1 expression. ROS production preceded androgen receptor protein loss and its down-regulation decreased when cells were treated with N-acetyl cysteine (an antioxidant).	[165]
Inorganic arsenic	normal human prostate stem-progenitor cells (PrSPCs)	Inorganic arsenic increased self-renewal and suppressed differentiation, activating the NRF2/KEAP1 pathway by inhibiting p62 degradation and increasing the expression of its downstream enzymes, such as NQO1, TXNRD1, GPX2, AKR1C3, HO-1 and UCHL1.	[173]
MnTE-2-PyP	normal human prostate fibroblasts	MnTE-2-PyP treatment of human prostate fibroblast cells in an irradiated hyperglycaemic environment protected against hyperglycaemia-induced cell death after radiation through the increase in NRF2 and NQO1 expression.	[176]
MnTE-2-PyP	mouse primary prostate fibroblast cells	MnTE-2-PyP increased NRF2, SOD2 and NQO1 expression, downregulating KEAP1 expression. The NRF2 pathway activation was essential to prevent myofibroblast formation and fibrosis during radiation exposure.	[177]
Clofibrate	DU145 cells	Clofibrate increased HO-1 expression, activating NRF2 signaling pathway, but not PPARα pathway. NRF2 silencing attenuated clofibrate-induced HO-1 gene transcription, while PPARα silencing had no effect on clofibrate-induced HO-1 expression.	[178]
Pterocarpanquinone	PC3, LNCaP, and LAPC4	Pterocarpanquinone increased apoptosis inducing ROS production and lipid peroxidation. It increased NRF2, NQO1 and SOD expression.	[183]
2-Chloroadenosine	PC3 and LNCaP cells	2-Chloroadenosine reduced viability and increased apoptosis. 2-Chloroadenosine reduced GSH content and increased ROS levels in PC3, whereas only ROS production was increased in LNCaP cells. 2-Chloroadenosine treatment increased NRF2 nuclear translocation in PC3 cells.	[187]
